# Case report: Diagnosis of ADCY5-related dyskinesia explaining the entire phenotype in a patient with atypical citrullinemia type I

**DOI:** 10.3389/fneur.2023.1266686

**Published:** 2023-11-09

**Authors:** Audrey Pontrucher, Magalie Barth, Alban Ziegler, Juan Manuel Chao de la Barca, Delphine Mirebeau-Prunier, Pascal Reynier, Chadi Homedan

**Affiliations:** ^1^Laboratoire de Biochimie et Biologie Moléculaire, Centre Hospitalier Universitaire, Angers, France; ^2^Service de Génétique, Centre Hospitalier Universitaire, Angers, France; ^3^Service de Génétique, CRMR AnDDI-Rares, CHU Reims, Reims, France

**Keywords:** ADCY5-related dyskinesia, adenylate cyclase type 5, argininosuccinate synthetase 1, citrullinemia type 1, ASS1

## Abstract

In this case study, we report the case of a 13-year-old girl with citrullinemia type 1 (MIM #215700), an autosomal recessive inherited disorder of the urea cycle, which was confirmed by the identification of a homozygous pathogenic variant in the argininosuccinate synthetase 1 (*ASS1*) gene. However, the patient presented abnormal hyperkinetic movements with global developmental delay and clinical signs that were not fully consistent with those of citrullinemia type 1 or with those of her siblings with isolated citrullinemia type 1. Exome sequencing showed the presence of a *de novo* heterozygous pathogenic variant in the adenylate cyclase type 5 (*ADCY5*) gene. The variant confirmed the overlap with the so-called ADCY5-related dyskinesia with orofacial involvement, which is autosomal dominant (MIM #606703), a disorder disrupting the enzymatic conversion of adenosine triphosphate (ATP) to cyclic adenosine monophosphate (cAMP). In addition to the citrullinemia-related low-protein diet and arginine supplementation, the identification of this second disease led to the introduction of a treatment with caffeine, which considerably improved the dyskinesia neurological picture. In conclusion, this case highlights the importance of clinical-biological confrontation for the interpretation of genetic variants, as one hereditary metabolic disease may hide another with therapeutic consequences.

**Summary:**

This article reports the misleading superposition of two inherited metabolic diseases, showing the importance of clinical-biological confrontation in the interpretation of genetic variants.

## Introduction

Citrullinemia type 1 (CTLN1) is an autosomal recessive urea cycle deficiency with an estimated prevalence of 1/250,000. It is caused by a pathogenic variants in the *ASS1* gene located in chromosome 9, which is responsible for deficiency in argininosuccinate synthetase (ASS1-D; MIM #215700), an enzyme of the urea cycle that produces argininosuccinate from citrulline and aspartate. This deficiency causes an accumulation of citrulline in biological fluids and deficiency in the urea cycle, resulting in neonatal hyperammonemia that may be life-threatening (1). It is also characterized by a high urinary excretion of orotic acid and a low argininemia level (2). There are two main forms of citrullinemia type 1: the classic acute neonatal form and the late-onset form (3). The clinical spectrum is a variable that includes, for the classical form during the newborn period, lethargy, drowsiness, feeding refusal, vomiting, and even lethal hyperammonemic encephalopathy. The late form may be asymptomatic or include milder delayed clinical signs related to the occurrence of transient episodes of hyperammonemia during increased protein catabolism. Clinical management consists of a low-protein diet, reducing protein catabolism, arginine supplementation, and nitrogen scavengers (4).

The *ADCY5* gene encodes adenylate cyclase type 5 (AC5), an enzyme involved in the conversion of adenosine triphosphate (ATP) to cyclic adenosine-3′,5′-monophosphate (cAMP). cAMP is a secondary messenger involved in many cellular signaling pathways (5). AC5 is primarily expressed in the striatum, so heterozygous pathogenic variants in the *ADCY5* gene affect the movement of regulatory systems (6). ADCY5-related dyskinesia with orofacial involvement, initially named “familial dyskinesia and facial myokymia,” is a rare hyperkinetic movement disorder. It is an autosomal recessive (MIM #619647) or dominant (MIM #606703) disorder. ADCY5-related dyskinesia is probably underdiagnosed due to a variety of clinical presentations and a large number of variants. Usually, it appears during childhood and manifests as paroxysmal dyskinesia associated with hyperkinetic movements with nocturnal predominance, chorea, dystonia, axial hypotonia, and myoclonus (7). There is currently no effective treatment, although the effects of caffeine are suggested to be effective (8).

We present here a case of ADCY5-related dyskinesia that was improved by caffeine in a young girl with citrullinemia type 1 whose clinical features were not fully compatible with this metabolic disease.

## Case report

**Figure 1:**
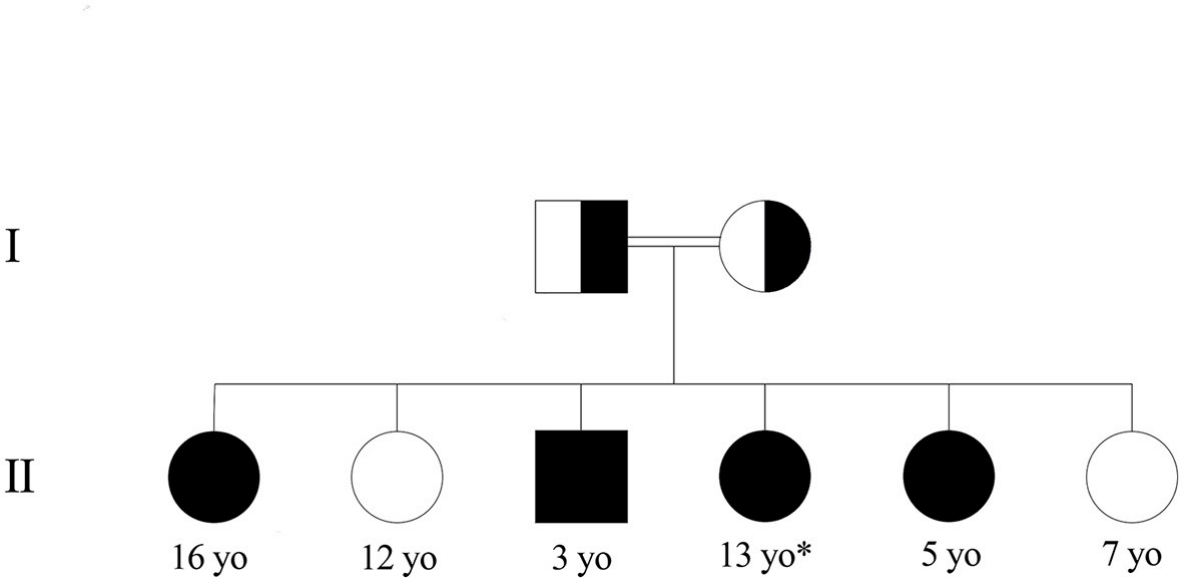
Family pedigree of the patient with type 1 citrullinemia. ^*^Patient. Healthy children had normal amino acid chromatography, and those with citrullinemia were confirmed with genetic testing.

The patient was a 13-year-old girl from Syria, born into a consanguineous family with third-degree-related parents (first cousins). She has an older sister, three younger sisters, and a younger brother (Figure 1). She lived in Lebanon for 6 years before arriving in France. At 8 months of age, she presented tremors, myoclonus, and dystonia of all four limbs. When she acquired the ability to walk at 14 months of age, she had instability and rigidity in all four limbs. She also had language delay. At 5 years of age, she was diagnosed with type 1 citrullinemia in Lebanon. She was prescribed arginine supplementation and a hypoprotein diet, which were poorly followed.

At 11 years of age, she was hospitalized in France for an etiological workup of her neurological disorder. On clinical examination, staturo-ponderal growth retardation (−3 DS), axial hypotonia with torticollis head bearing, dystonia during sleep, ataxic gait, frequent falls, orofacial dyskinesia, and intellectual and language development delay with dysarthria were noted. A protein-loading test was performed, showing a normal ammonia cycle under 2 g/kg/days of protein. Analysis of plasma amino acids showed moderate elevations of citrulline (550 μM/L, *N* < 33 μM/L) and glutamine (690 μM/L, 486 < *N* < 670 μM/L) with a decrease in arginine (50 μM, 57 < *N* < 97 μM/L). Urinary orotic acid, a specific metabolite in urea cycle deficiency, was elevated (47 μmol/mmol creatinine, *N* < 6 μmol/mmol of creatinine). Hepatic function and abdominal ultrasound were normal. The study of the panel of genes involved in the urea cycle revealed the homozygous missense variant NM_054012.4(ASS1):c.535T>C (p.Trp179Arg) in the *ASS1* gene, reported as pathogenic by multiple submitters in ClinVar (PS3, PP5, PP3, PM3, and PM2 ACMG criteria) (9) (https://www.ncbi.nlm.nih.gov/clinvar/variation/6335), confirming the diagnosis of CTLN1. A 1 g/kg/day protein diet and arginine supplementation were implemented. Then, systematic family screening was performed, revealing familial citrullinemia with three out of four siblings affected.

Although the symptoms were initially attributed to citrullinemia, this was reconsidered because of the discrepancies between the clinical picture and typical signs of citrullinemia. Indeed, citrullinemia alone could not explain the clinical features, especially the hyperkinetic movement disorder in the patient. Moreover, the siblings with citrullinemia did not present similar symptoms in the absence of acute decompensation, so further investigations were performed. Given the history of citrullinemia, we performed an MRI of the brain to ensure that this was not dystonia sequelae of previous decompensation. Once the MRI was normal, we did a dystonia workup, looking, in particular, for neurotransmitter abnormalities. As the cerebrospinal fluid was normal (neurotransmitters, amino acid chromatography, lactate, pyruvate, glycorachia, and proteinorachia), whole-exome sequencing was performed. Brain MRI and lumbar puncture results (neurotransmitters, amino acid chromatography, lactate, pyruvate, glycorachia, and proteinorachia) were normal. First, whole-exome sequencing was performed in search of a recessive pathogenic variant because the consanguinity was negative. However, it revealed a heterozygous *de novo* missense pathogenic variant in exon 2 of the *ADCY5* gene [NM_183357.3:c.1253G>A; p.Arg418Gln, ClinVar file: https://www.ncbi.nlm.nih.gov/clinvar/variation/218354/?oq$=$%22NM_001199642.1:c.203G%3eA%22%5bvarname%5d&m$=$NM_183357.3(ADCY5):c.1253G%3eA%20(p.Arg418Gln)]. This variant acts as a gain-of-function (10). It was absent in the gnomAD population database and was reported as pathogenic in the ClinVar database, and was found to be *de novo* and classified as pathogenic according to the ACMG classification (PM2, PP5, PS3, and PS2).

A caffeine-based treatment was implemented. It is usually recommended to start with half a cup, then a full cup, and then more as it progresses. A 3-day inpatient introduction test was conducted to verify the absence of side effects. Because she disliked the flavor of coffee, we made pharmaceutical preparations of caffeine at 50 mg/capsule, which she takes three times a day (150 mg/day). After treatment for 3 months, the patient showed significant improvement in the quality of life, with a reduction in hypotonia, tremors, myoclonus, and dystonia and a clear improvement in dysarthria and fatigability.

## Discussion

Some missense pathogenic variants in the *ASS1* gene are attributable to mild, moderate, or even asymptomatic forms of CTLN1. In these cases, patients present biological signs instead of clinical signs. The homozygous *ASS1* c.535T>C variant (p.Trp179Arg) identified in the patient has been described as being responsible for moderate forms of type 1 citrullinemia, often asymptomatic (11). This pathogenic variant is geographically mainly present in Eastern Europe and, more particularly, in Turkey (12). Unlike other pathogenic variants leading to a complete enzymatic deficiency of argininosuccinate synthase that is responsible for more severe forms, the p.Trp179Arg variant induces a partial enzymatic deficiency of arginosuccinate synthase with an alteration in the substrate binding site, thus decreasing its affinity for aspartate (3). Residual enzyme activity was estimated to be 6% of the wild type (13). This residual activity would be sufficient to remove toxic ammonia, resulting in moderate forms of citrullinemia. Nevertheless, the clinical phenotype remains difficult to predict. Some prognostic markers are being studied, such as the level of citrullinemia, molecular biology, and residual enzymatic activity (14, 15). However, a high level of citrulline (>2,000 μM), which is more frequently found in classical forms of citrullinemia, can also be found in asymptomatic forms (2, 16). There is intrafamilial phenotypic variability in citrullinemia, and its discovery in the patient led to the diagnosis of the asymptomatic siblings, and the management consisted of limiting protein intake, supplementing with arginine, and closely monitoring for decompensation risk. However, good neurocognitive development in children has been described in families with the p.Trp179Arg variant without the use of a specific diet (15). The need for a special diet in these moderate forms remains uncertain.

The patient presented with abnormal hyperkinetic movements with global developmental delay and clinical signs that were not consistent with those of moderate type 1 citrullinemia or with those of her siblings with asymptomatic CTLN1. Even if there is intrafamilial phenotypic variability, the significant difference in clinical presentation between members of the same family should lead to a search for additional causes of disease. Further investigations were then performed. Whole-exome sequencing revealed another heterozygous *de novo* missense pathogenic variant in the *ADCY5* gene. This isoform is expressed primarily in the striatum, a component of the motor nervous system that enables voluntary motor activity and prevents involuntary movements (6). The striatum consists mainly of GABAergic medium spiny neurons, which are stimulated by glutaminergic transmission and regulated by the dopaminergic system. Type 1 (D1) dopamine receptors have a stimulatory effect, while type 2 (D2) receptors have a more inhibitory effect. The majority of pathogenic variants in the *ADCY5* gene are gain-of-function mutations (10), resulting in increased ATP affinity and inhibition of cAMP-degrading phosphodiesterases. Thus, the level of cAMP increases, leading to hyperactivation of downstream signaling pathways and dysregulation of the middle spiny neurons, resulting in movement disorders called dyskinesia with orofacial involvement. The clinical picture is heterogeneous and may include paroxysmal dyskinesia, chorea, myoclonus, dystonia, tremor, axial hypotonia, and involuntary movements, mainly in the face, neck, and arms. A global developmental delay is associated, including language disorders, with ongoing dysarthria (7, 17, 18). Over time, the frequency and severity of dyskinesia episodes may stabilize or increase with a paroxysmal exacerbation of hyperkinetic movements (https://www.ncbi.nlm.nih.gov/books/NBK263441/).

Effective treatment and recommendations are lacking. Deep brain stimulation may be proposed alongside anticonvulsant benzodiazepines (clonazepam). A new caffeine-based treatment is one of the proposed treatments (8, 19). Indeed, caffeine is an antagonist of A2A receptors. The latter, stimulating AC5, is preferentially located in striatal neurons that express D2 receptors (20). D2 and A2A are G-protein-coupled receptors with allosteric interactions between them. Thus, antagonizing A2A receptors potentializes the effects of D2 receptors, reinforces the dopamine inhibitory pathway, and decreases ADCY5 activity and the occurrence of dyskinesia (5).

A retrospective study on the efficacy of caffeine in the management of ADCY5-related dyskinesia reported that 87% of patients respond to caffeine (21). The primary endpoint was an improvement in global involuntary movements, which was measured to be at least 40%. This study highlighted that caffeine is an effective and well-tolerated treatment for most patients with ADCY5-related dyskinesia. An improvement in terms of frequency and duration of movement disorders was highlighted, especially walking, dysarthria, hypotonia, and quality of life. Moreover, even when taken in the evening, no sleep disturbance due to caffeine consumption was reported. However, the adjustments in terms of dosage and intervals of intake remain to be defined and are currently based on personalized management.

This case shows that we should not always stop at the early primary diagnosis of an inherited metabolic disorder. Even when such a disease is confirmed biochemically and genetically, the consistency of the clinical picture with the molecular diagnosis remains paramount because the entire phenotype is not always explained by only one disease. The diagnosis of this second genetic disease was essential for the health of the patient since it allowed a considerable improvement in the symptoms under treatment with caffeine. Furthermore, in the absence of corresponding data in the literature, it would be interesting to search whether there could be a pathophysiological interaction between the toxicity of hyperammonemia associated with citrullinemia and the gain-of-function of AC5 in ADCY5-related dyskinesia on the striatal function.

## Data availability statement

The datasets presented in this article are not readily available because of ethical and privacy restrictions. Requests to access the datasets should be directed to the corresponding author.

## Ethics statement

Ethical review and approval were not required for this case report in accordance with the local legislation and institutional requirements. Written informed consent from the patients/participants legal guardian were obtained for genetic analyses in accordance with the national legislation and the institutional requirements. Written informed consent was obtained from the patient's legal guardian/next of kin for the publication of any potentially identifiable images or data included in this article.

## Author contributions

AP: Conceptualization, Writing—original draft, Writing—review & editing, Data curation, Formal analysis, Investigation. MB: Conceptualization, Data curation, Formal analysis, Investigation, Writing—review & editing, Supervision. AZ: Data curation, Formal analysis, Investigation, Writing—review & editing, Methodology. JC: Formal analysis, Investigation, Writing—review & editing. DM-P: Formal analysis, Investigation, Writing—review & editing. PR: Writing—review & editing, Conceptualization, Project administration, Supervision, Validation, Writing—original draft. CH: Conceptualization, Data curation, Formal analysis, Investigation, Methodology, Project administration, Supervision, Writing—original draft, Writing—review & editing.
